# Evaluation of the nutrition knowledge of sports department students of universities

**DOI:** 10.1186/1550-2783-8-11

**Published:** 2011-09-05

**Authors:** Yahya Ozdoğan, Ayse Ozfer Ozcelik

**Affiliations:** 1 Department of Nutrition Sciences, School of Home Economics, Ankara University, Ankara, Turkey; 2 Department of Nutrition and Dietetics, Faculty of Health Sciences, Ankara University, Ankara, Turkey

## Abstract

**Background:**

Individuals who have knowledge on the importance of adequate and balanced diet and reflect this knowledge to their behaviors are considered to be more successful in sports life. The present study aims to evaluate the nutrition knowledge of students receiving sports education in universities.

**Methods:**

The study sample consists of 343 voluntary students from the Sports Departments of Hacettepe, Gazi and Ankara Universities in Ankara. The questionnaire used in the study included a demographic section, and 30 questions on true-false nutrition knowledge. For the reliability of the questionnaire, the internal consistency coefficient was calculated and the Kuder Richardson (KR-20) value was found to be 0.71. For higher reliability, 9 dysfunctional questions were excluded from the questionnaire. The research data were collected through a questionnaire form and face-to-face interviews. For the statistical analyses of the data, tables were prepared to show mean, standard deviation ($\bar{X}\pm SD$) and percentage (%) values. In order to determine the nutrition knowledge of students, the "independent t test" was used for nutrition lesson and gender.

**Results:**

University students receiving sports education and expected to continue their professional lives on sport-related fields were determined to have the lack of knowledge on nutrition. The mean value about the nutrition knowledge of the first year students was found 11.150 ± 2.962, while the mean value of the fourth year students was 13.460 ± 3.703, and the difference is statistically significant (p = .000).

**Conclusion:**

Students, coaches and teachers in physical education were found not to give the necessary importance to their diets, and they were still not aware of the importance of nutrition on performance.

## Background

The study of nutrition dates back to over 200 years; however, sports nutrition is relatively a new discipline involving the application of nutritional principles to enhance the athletic performance. Nutrition affects a sportsman in many ways. At the basic level, it plays an important role in achieving and maintaining health. Optimal nutrition can reduce fatigue, allowing a sportsman to train and compete longer or recover faster between training sessions [1]. Nutrition is an important component of any physical fitness program. The main dietary goal of active individuals is to obtain adequate nutrition to optimize their health and fitness or sports performance [2].

Indeed, nutrition affects almost every process in the body involved in energy production and recovery from exercise. To understand and apply the principles of sport nutrition, some basic understanding of nutrition is necessary. This includes the knowledge of biochemical and physiological processes that occur in different cells and tissues as well as how these processes are integrated throughout the body [3].

There are many reasons why nutritional advice is not followed. It may be due to the lack of knowledge or information, and interest of making a change in one's diet, or certain perceived or encountered barriers that may prevent people from eating healthier diets such as the lack of money (cost), lack of time (too busy with work) or taste [4]. Athletes may often rely on coaches for nutrition guidance in certain sports. Therefore, when coaches are misinformed about nutrition, this becomes a potential problem for athletes, as well [5]. Nutrition training can be conveyed to the individuals through regular and wide educational programs as well as the individual training himself on his own settings [6]. Various studies focused on the necessity of nutrition training [7-9].

Prospective teachers and coaches receiving education at higher schools of sports increase their knowledge on nutrition and transfer their knowledge to next generations. Therefore, the quality of the education they receive is especially important. This study aims to investigate the nutrition knowledge of students receiving sports education in universities.

## Methods

### Subjects

The study sample includes the first- (n: 260) and fourth-year (n: 345) students attending the sports teaching and coaching department of Hacettepe, Ankara, and Gazi Universities. These universities offer corresponding courses on nutrition. In total, the study was carried out with 343 voluntary students, 180 from the first year students (69.2%) and 163 (47.2%) from the fourth year students.

### Procedure

In this descriptive study, a questionnaire form was developed to evaluate the nutrition knowledge of students receiving sports education at universities. The questionnaire form was composed of two sections: the first part was designed to obtain information about the demographic characteristics of the students, while the second part contained statements related to nutrition knowledge (Appendix A). No ethical approval is needed for a questionnaire in Turkey.

In order to evaluate the knowledge on nutrition, the participant students were given 30 statements which could be replied as "true" or "false". An instrument was developed using carefully selected questions from questionnaires created by Rosenbloom et al., Zawila et al., Juzwiak and Ancona-Lopez and Ersoy [7,8,10,11]. The research data were collected through a questionnaire and face-to-face interviews.

### Statistical Analysis

After administering the questionnaire to the individuals and assessing it, a reliability test was applied. "Kuder Richardson", the internal consistency coefficient, was calculated for the reliability of questionnaire, and the KR-20 value was found to be 0.71. For higher reliability, 9 dysfunctional questions were excluded from the 30-items questionnaire (Appendix B) and the questionnaire was evaluated considering the remaining 21 items. Accordingly, the "nutrition knowledge" scale was concluded as a reliable instrument.

In the evaluation of nutrition knowledge, each correct answer was given 1 point, whereas no point was given to wrong answers. Nutrition knowledge was evaluated using a questionnaire form consisting of 21 questions in terms of taking or not taking nutrition lesson (1^st ^year -the ones who did not take nutrition lesson, 4^th ^year - the ones who took nutrition lesson). The data of the study were evaluated using SPSS 16.0 package program. The nutrition knowledge of students was examined by gender and class variables. For the statistical analyses of the data, tables were prepared to show mean, standard deviation ($\bar{X}\pm SD$) and percentage (%) values. Nutrition knowledge score was dependent variable in the study, while gender and grade were independent variables. To determine the nutrition knowledge of students, the "independent t test" was used for nutrition lesson and gender. A criterion alpha level of < 0.05 was used to determine statistical significance.

## Results

### Descriptive Data

Participants were composed of males (60.3%) and females (39.7%). In the general sample, the mean age was 22.19 ± 2.76 years, while the mean age of females was 21.33 ± 2.09 and the mean age of males was 22.76 ± 2.99. The majority of the students (68.6%) were determined to live with their families, while others live in student residence (22.1%), with their friends (5.5%), alone (2.9%) and in the sport facility they were working (0.9%). Most of the students (64.7%) stated to be interested in active sports, while the rest (35.3%) did not actively make sports. Nearly half of the students actively making sports (55.8%) were interested in team sports, while the other half of them were interested in endurance sports (18.9%), sports requiring immediate strength (15.4%), and combat sports (9.9%).

### Nutrition knowledge score

The mean nutrition knowledge scores, standard deviation and t-test results of the students are presented in Table 1 according to the variables of taking nutrition lesson and gender.

**Table 1:** Students' mean nutrition knowledge scores according to the variables

Variables	n	$\bar{X}$	SD	df	t	p
Grade						
First	180	11.150	2.962	341	6.406	.000*
Fourth	163	13.460	3.703			
Gender						
Female	136	11.985	3.446	341	1.118	.264
Male	207	12.420	3.573			
Total	343	12.247	3.525			

*p < 0.001

The mean nutrition knowledge score in the general sample was 12.247 ± 3.525. When the mean knowledge scores were examined, it was determined that the fourth year students (13.460 ± 3.703) got higher scores than the first year students (11.150 ± 2.962); in addition, males (12.420 ± 3.573) got higher scores than females (11.985 ± 3.446). The difference between the knowledge scores of the first year students and fourth year students was found to be statistically significant (p = .000).

## Discussion

The present study investigated the nutrition knowledge of students receiving sport education in universities considering whether they took nutrition class (1^st ^and 4^th ^year students) and gender. Most of the participant students were both continuing their university education and pursuing sports life in various clubs. In addition to the energy and food elements needed by regular university students, there are some extra requirements for sports type of these students. It is considered that people with inadequate knowledge of nutrition will also be unaware of additional nutrient needs.

Over half of the participants (56.3%) correctly answered the statement "eating carbohydrates makes you fat" as false. In another study, the majority of males (74.0%) and females (75.0%) correctly answered the same statement. The response to the statement of carbohydrates and the relationship between carbohydrates and body fat are encouraging, as many believe that those trying to improve body composition should avoid carbohydrates [7].

The sportsmen are inclined to think that sweets would provide quick energy just before competition. This prejudice may lead to rely on candy to provide the energy that should come from complex carbohydrates. The underlying goal of eating candies before exercise is to boost energy and minimize insulin surge that transports sugar out of the bloodstream into the muscles. Simple sugars induce high insulin, and when used before exercise, this can lower the blood sugar and elicit the fatigue as well as lightheadedness associated with hypoglycemia [11,12]. A big proportion of the students (72.3%) correctly answered the statement "basic sugars like cube sugar, jam, and honey are the most suitable energy sources for sportsmen" as false.

Carbohydrates are the source of muscle energy followed by fats and proteins, whereas vitamins, minerals, and water are also essential for health but do not provide energy [13]. It is important for athletes to consume enough carbohydrates to maintain blood glucose and to replace glycogen stores [14,15]. Over half of the participants (61.2%) correctly answered the statement "glycogen muscles store carbohydrate". In a study carried out by Juzwiak and Ancona-Lopez [10], an important part of the participants (74.0%) gave correct answers to the statement "glycogen levels (stored carbohydrate) can affect the energy level available for exercise".

The majority of the participant students (77.8%) answered the statement "protein is the main energy source for the muscle" as false. In the previous studies on this matter, the rates of people with correct knowledge changed between 28.0% and 54.0% [7,8,10,16]. The athletes should be informed about the fact that proteins are not the main energy source for the muscles. The adequate energy must be consumed in the form of carbohydrate to replenish glycogen stores [5].

The general consensus among nutritionists is that calories from fat should be maintained at approximately 30% of energy intake [17]. There is no benefit for athletes in fat intake less than 15% or greater than 30% of total calories [18]. A significant proportion of the participants (78.4%) correctly answered the statement "fats have important roles in the body". Body fats have many functions like providing fuel to most tissues, working as an energy reserve, insulating the body and nerve fibers, supporting and protecting vital organs, lubricating body tissues, and creating an integral part of cell membranes [19].

Iron plays an important role in exercise as it is required for the formation of hemoglobin and myoglobin, which bind oxygen in the body, and for enzymes involved in energy production. Iron depletion (low iron stores) is one of the most prevalent nutrient deficiencies observed in athletes, especially in female athletes [18]. Many female athletes and nonathletes consume inadequate amounts of iron [20]. Over half of the participants (65.9%) correctly answered the statement "Iron-deficiency anemia results in a decrease in the amount of oxygen that can be carried in the blood". Athletes should be screened periodically to assess iron status. Changes in iron storage (low-serum ferritin concentrations) occur first, followed by low-iron transport (low- serum iron concentrations), and eventually result in iron deficiency anemia [18].

While the absorption ratio of iron in plant food is around 4-15%, it is 25-30% in meat [21]. In the present study, more than half of the subjects (65.3%) answered the statement "iron in meat is absorbed at the same rate as iron in a plant food" as false. Over half of the students (67.6%) correctly answered the statement "the body can synthesize vitamin D upon exposure to the sun". The two primary sources of vitamin D are fortified foods like milk, and ultraviolet conversion in the skin, which produces the vitamin [14].

Over half of the students (67.9%) correctly answered the statement "vitamin supplementation is recommended for all physically active people" as false. The reason why the students could not answer the statement correctly at higher rate can be attributed to the common idea that additional vitamin and minerals are useful. In a similar study, the rate of participants giving the same answer was found lower (10.0%) [8]. Athletes will not need vitamin-mineral supplements if they consume adequate energy from a variety of foods to maintain body weight [14,18]. A recent study has shown that the majority of college athletes (88.0%) used one or more nutritional supplements [22]. A smaller part of the participants (12.8%) answered the statement "skipping meals is justifiable if you need to lose weight quickly" as true. This indicated that skipping a meal was generally considered enough to lose weight. This situation demonstrates the fact that sportsman students should review their knowledge on nutrition. In a study carried out with adolescents and young male hockey players, a significant part of the participants (84.0%) stated that skipping meal was not a good way to lose weight [10].

The micronutrients vitamins and minerals also have an important role in the health of athletes. They are essential players in energy production, hemoglobin synthesis, bone health, immune function, and antioxidant activity [18]. More than half of participants (64.1%) correctly answered the statement "vitamins are good sources of energy" as false. In the previous studies, the rate of people having the correct knowledge on this matter was quite low [8,16,23]. Especially, the statements related to nutritional contents were answered at lower rates, which demonstrated the insufficiency of the education on nutrition or the short retention periods of education. Students did not have sufficient knowledge on nutrition, which was one of the main reasons affecting the performance of sportsmen; for this reason, the education system should be reviewed in this regard.

Food that is easily digested and absorbed by body should be preferred soon after the training. This includes fruit, bread, cereal, skimmed milk, yoghurt, juice, and sports drinks which are richer than carbohydrate and include low fat. On the other hand, some other foods including coke, chocolate, biscuits, chips, and lait crémeux should not be consumed as they are flatulent and remain in the stomach for a long time [11]. Only a small proportion of the participant (25.1%) students answered that "the food like chocolate, biscuit and chips are not appropriate for consuming after the training". This indicated that students did not have enough knowledge about the food they consumed after the training.

Timing of food consumption based on the time of a competition or exercise event is important. The ability to perform and recover from exercise can be positively or negatively affected by dietary intake before, during, and after the event. The pre-event meal should be low in fat, fiber, and caffeine; moderate in protein; and high in complex carbohydrates and fluid. Meals are best consumed at least 3-4 hours before the competition to minimize gastric distress, nausea, vomiting, cramps, and sluggishness [13]. The majority of the students (81.6%) correctly answered the statement "the last meal should be consumed 3-4 hours before the competition".

Over half of the students (66.8%) correctly answered the statement "bananas are good sources of potassium". Potassium is a cation, and the major intracellular electrolyte. It is the third most abundant mineral in the body and a component of muscle. Potassium is also needed for the maintenance of fluid balance [20]. There is 370 mg potassium in 1000 g banana [24]. A small part of the participants (14.9%) correctly answered the statement "males and females in the same age group spend the same amount of calorie during the same exercise". The regular functions of body like keeping the body warm and regulating the movements are ensured by proper amounts of energy intake. The energy requirement differs among conditions such as age, gender, body combination, body frame, temperature of the environment and diseases [25]. The low rate of correct answers for this statement demonstrated that the difference between gender was disregarded, which could be caused by lack of knowledge.

As the sodium naturally found in the vegetables and cereals provides the daily requirement, there is no need to add extra salt except for special conditions. From this regard, less than half of the participants (37.6%) correctly answered the statement "salt is an essential part of a healthy diet" as false. Salt also has adverse effects on health, increasing blood pressure and causing edema in body. Therefore, salt consumption should be restricted. Calcium is especially important for the building and repair of bone tissue and the maintenance of blood calcium levels. Inadequate dietary calcium increases the risk of low bone mineral density and stress fractures [18]. The majority of the students (81.5%) correctly answered that "milk and milk products are the best sources of calcium". The high rate of correct answers indicated that the students were aware of the importance of calcium. In a study with female athletes, nearly all of the participants (92.0%) were found to know this fact which was consistent with the findings of the present study [26].

Water is the most necessary nutrient for the body and it must be kept available at all times during the practice and competition [12]. An athlete loses too much water due to dehydration and may have low performance and high risk of heat stroke [27]. Water consumption is important for sportsmen and it was questioned with the statement of "dehydration decreases performance", which was correctly answered by only 43.1%. In the study performed by Rosenbloom et al. [7], the rate of people having knowledge on this matter was more than twice as much as the rate determined in the present study. An important part of the participants (69.7%) correctly answered the statement "during the activity, feeling thirsty is an enough indicator of the need for liquid" as false. In a similar study, this ratio was 66.0% [10]. It is important for athletes to consume enough fluids throughout the day, during exercise and recovery periods of exercise [5,12].

More than two third of the fat should be in unsaturated forms. Because saturated fat is associated with heart disease, it is wise to reduce the saturated fat intake. Foods high in saturated fats are of animal origin in general and include red meat and whole milk. Unsaturated fats are typically oils and soft or liquid at room temperature [12]. The amount of saturated fats in daily diet should be decreased and unsaturated fats should be cautiously consumed; however, only a small part of students (32.7%) correctly answered the false statement that "saturated and unsaturated oils both have equal effect on health".

An important proportion of the students (67.1%) correctly answered that "alcohol consumption can affect absorption and utilization of nutrients". Many alcoholics are malnourished, either because they ingest too little essential nutrients (e.g., carbohydrates, proteins, and vitamins) or because alcohol and its metabolism prevent the body from properly absorbing, digesting, and using those nutrients [28].

In this study, the highest score was 21 which could be obtained when all the questions were correctly answered. However, the mean score of the participants was 12.247 ± 3.525, which was considered low and indicated the inadequacy of nutrition knowledge of students. In various studies, sportsmen's nutrition knowledge was also reported inadequate [7,22,26,29-33]. On the other hand, there were some other studies determining nutrition knowledge adequate [10,34]. Considering the importance of nutrition for sportsmen, it is necessary to increase the knowledge of sportsmen and their trainers on nutrition.

In this study, it was found that the mean knowledge scores of the male students were higher compared to female students. However, the difference was not statistically significant (p > 0.05). In other studies carried out by Rosenbloom et al. and Corley et al. [7,34], it was determined that the nutrition knowledge did not vary according to gender. In contrast, there were some other studies reporting that the knowledge levels of females were higher than males [31,35]. This discrepancy might be caused by the difference between the study groups. The mean nutrition knowledge scores of the fourth year students were higher than those of the first year students.

The difference between the first and fourth year students was found statistically significant (p < 0.001). Considering the fact that the fourth-year students took nutrition class, the importance of this information could become more evident. This was caused by the lack of knowledge. Increasing the education on nutrition will also increase the knowledge scores on this matter. Nutrition education should be more emphasized and the permanency of the education should be provided.

## Conclusions

In general, neither athletes nor coaches have sufficient knowledge on nutrition to create an environment that can result successfully in enhanced performance and optimal health [5]. The importance of nutrition education is increasingly recognized at present, and there is a consensus that people's food choices, dietary practices, and physical activity behaviors influence health [36]. Nutrition knowledge was found low for the students enrolled in universities to become prospective teachers and coaches and they were not aware of the importance of the nutrition for performance. Enough and balanced nutrition should be a perfect life style and an eating habit for a sportsman. The number of courses related to nutrition should be increased in universities and the main objective in these courses should be to make the theoretical knowledge applicable in daily life. Experienced sportsmen and trainers should pursue ways to educate young people on how to select nutritious foods that will promote a lifetime of good health [12]. Further studies evaluating the nutrition knowledge of amateur-professional sportsmen, coaches, and even the people living with them might be useful.

## Competing interests

The authors declare that they have no competing interests.

## Authors' contributions

AOO wrote the analysis plan with input from other author and drafted the manuscript, YO conducted the analysis and participated in the interpretation of the results and provided critical comments. Both authors were involved in the implementation of the study as well as read and approved the final manuscript.

## Appendix A. Items selected for the questionnaire

Statements

4 Protein is the main energy source for the muscle (F)

6 Fats have important roles in the body (T)

7 Iron-deficiency anemia results in a decrease in the amount of oxygen that can be carried in the blood (T)

8 Iron in meat is absorbed at the same rate as iron in a plant food (F)

9 The body can synthesize vitamin D upon exposure to the sun (T)

10 Vitamin supplementation is recommended for all physically active people (F)

11 During the activity, feeling thirsty is an enough indicator of the need for liquid (F)

12 Skipping meals is justifiable if you need to lose weight quickly (F)

14 The food like chocolate, biscuits, chips are the most appropriate foods to be consumed soon after the training (F)

15 Vitamins are good sources of energy (F)

17 Alcohol consumption can affect absorption and utilization of nutrients (T)

19 Saturated and unsaturated oils both have the equal effect on the health (F)

21 Eating carbohydrates makes you fat (F)

22 Dehydration decreases performance (T)

23 The last meal before a competition should be consumed 3-4 hours before the competition (T)

25 Males and females at the same age group spend equivalent amount of calorie during the same exercise (F)

26 Bananas are good sources of potassium (T)

27 Salt is an essential part of a healthy diet (F)

28 Milk and milk products are the best sources of calcium (T)

29 Basic sugars like cube sugar, jam, honey are the most suitable energy sources for sportsmen (F)

30 Glycogen muscles store carbohydrate (T)

Note: (T) = true, (F) = false.

## Appendix B Items excluded from the questionnaire

1 Equivalent weights of carbohydrate and protein have approximately the same caloric value (T)

2 A slice of bread is an example of one serving from the bread and cereals food group (T)

3 Protein is not stored in the body; therefore, it needs to be consumed every day (T)

5 No more than 15% of calories in the diet should be provided by fat (F)

13 Caffeine has been shown to improve endurance performance (T)

16 Fiber in the diet may help to decrease constipation, decrease blood cholesterol levels, and prevent cancers (T)

18 When trying to lose weight, acidic food such as grapefruit is of special value because it burns fat (F)

20 Carotenoids help to prevent the formation of free radicals (T)

24 Sports drinks are better than water (T)

Note: (T) = true, (F) = false.
